# Evaluation of AMSTAR to assess the methodological quality of systematic reviews in overviews of reviews of healthcare interventions

**DOI:** 10.1186/s12874-017-0325-5

**Published:** 2017-03-23

**Authors:** Michelle Pollock, Ricardo M. Fernandes, Lisa Hartling

**Affiliations:** 1 Alberta Research Centre for Health Evidence, Department of Pediatrics, University of Alberta, Edmonton, Canada; 2 Clinical Pharmacology Unit, Instituto de Medicina Molecular, University of Lisbon, Lisbon, Portugal; 3 Department of Pediatrics, Santa Maria Hospital, Lisbon, Portugal; 4 4-472 Edmonton Clinic Health Academy, 11405 87 Avenue NW, Edmonton, AB T6G-1C9 Canada

**Keywords:** Systematic review, Overview of reviews, Knowledge synthesis, Methodological quality, Observer agreement, Bias

## Abstract

**Background:**

Overviews of reviews (overviews) compile information from multiple systematic reviews (SRs) to provide a single synthesis of relevant evidence for decision-making. It is recommended that authors assess and report the methodological quality of SRs in overviews—for example, using A MeaSurement Tool to Assess systematic Reviews (AMSTAR). Currently, there is variation in whether and how overview authors assess and report SR quality, and limited guidance is available. Our objectives were to: examine methodological considerations involved in using AMSTAR to assess the quality of Cochrane and non-Cochrane SRs in overviews of healthcare interventions; identify challenges (and develop potential decision rules) when using AMSTAR in overviews; and examine the potential impact of considering methodological quality when making inclusion decisions in overviews.

**Methods:**

We selected seven overviews of healthcare interventions and included all SRs meeting each overview’s inclusion criteria. For each SR, two reviewers independently conducted AMSTAR assessments with consensus and discussed challenges encountered. We also examined the correlation between AMSTAR assessments and SR results/conclusions.

**Results:**

Ninety-five SRs were included (30 Cochrane, 65 non-Cochrane). Mean AMSTAR assessments (9.6/11 vs. 5.5/11; *p* < 0.001) and inter-rater reliability (AC1 statistic: 0.84 vs. 0.69; “almost perfect” vs. “substantial” using the Landis & Koch criteria) were higher for Cochrane compared to non-Cochrane SRs. Four challenges were identified when applying AMSTAR in overviews: the scope of the SRs and overviews often differed; SRs examining similar topics sometimes made different methodological decisions; reporting of non-Cochrane SRs was sometimes poor; and some non-Cochrane SRs included other SRs as well as primary studies. Decision rules were developed to address each challenge. We found no evidence that AMSTAR assessments were correlated with SR results/conclusions.

**Conclusions:**

Results indicate that the AMSTAR tool can be used successfully in overviews that include Cochrane and non-Cochrane SRs, though decision rules may be useful to circumvent common challenges. Findings support existing recommendations that quality assessments of SRs in overviews be conducted independently, in duplicate, with a process for consensus. Results also suggest that using methodological quality to guide inclusion decisions (e.g., to exclude poorly conducted and reported SRs) may not introduce bias into the overview process.

**Electronic supplementary material:**

The online version of this article (doi:10.1186/s12874-017-0325-5) contains supplementary material, which is available to authorized users.

## Background

Systematic reviews (SRs) aim to answer a specific clinical question by identifying, selecting, appraising and synthesizing all relevant primary studies using explicit and well-defined methods [1]. The number of published SRs is constantly increasing [2]. To help manage this information overload, overviews of reviews (overviews) have emerged as an increasingly popular knowledge synthesis product. Overviews use explicit and systematic methods to integrate information from multiple related SRs to provide a comprehensive synthesis of all SR evidence related to a specific clinical question [3]. As a result, overviews are broader in scope than any individual SR, and often examine evidence from multiple SRs to assess the efficacy or effectiveness of multiple interventions for preventing or treating one specific clinical condition. Overviews can include both SRs published in and outside of the *Cochrane Database of Systematic Reviews* (CDSR; referred to as “Cochrane SRs” and “non-Cochrane SRs”, respectively). An estimated 48-86% of published overviews include both Cochrane and non-Cochrane SRs, while the remaining overviews include Cochrane SRs only [4–6].

There is consensus in the research community that researchers conducting overviews of healthcare interventions ought to assess and report the methodological quality of the SRs included in their overview [7]. These assessments should ideally be conducted by two independent reviewers, with a process for consensus, and reported transparently [3, 7]. However, researchers conducting overviews have indicated that assessing methodological quality of SRs may be difficult and time-consuming [7]. Studies have indicated that only 37-64% of published overviews assess and report the methodological quality of their included SRs, and among these overviews, there is variation in the methods used [4–6]. This variation is not surprising, as to date there is limited guidance regarding the specific methods that should be used to assess SR quality.

Quality assessments of SRs are important in overviews for two main reasons. First, quality assessments should be used by overview authors when making conclusions in overviews (e.g., to help contextualize the evidence by providing insight into whether and to what extent SR methods may have affected the comprehensiveness and results of overviews). However, it is not known whether and how existing quality assessment criteria need to be modified for use in overviews [7]. Assessing the quality of SRs in the context of overviews may pose unique challenges, and decision rules may be helpful to promote consistent assessments both within and across overview topics. Second, results of quality assessments may help inform inclusion decisions [7]. This may be especially relevant when including non-Cochrane SRs in overviews. On average, non-Cochrane SRs have lower methodological rigor than Cochrane SRs, and the methods and reporting of non-Cochrane SRs can vary widely [8–10]. Researchers conducting overviews have indicated that including lower-quality SRs in overviews can increase the complexity of the overview process because data may be missing, poorly reported, or inconsistently reported in the SRs, and it is unclear what to do in these situations (e.g., should overview authors refer back to the relevant primary studies, or attempt to use the poorly conducted and/or reported SRs?) [7]. However, existing methodological guidance on this topic is conflicting [7]. One potential solution proposed by researchers [7] and employed by overview authors [11–18] is to use the results of methodological quality assessments to identify and exclude SRs with gross deficiencies in conduct and/or reporting that would be difficult to include and use in overviews. However, using results of quality assessments to inform inclusion decisions may introduce bias if the results and conclusions of these SRs differ systematically from other well-conducted and reported SRs.

A MeaSurement Tool to Assess systematic Reviews (AMSTAR) is the most frequently mentioned tool for assessing SR quality in overviews [7]. AMSTAR consists of eleven questions designed to assess the appropriateness of the methods used at different stages of the SR process, and it has been shown to be reliable, valid, and easy to use when assessing the quality of published SRs [19–21]. The objectives of the present study were: 1) to examine methodological considerations involved in using the AMSTAR tool to assess the quality of Cochrane and non-Cochrane SRs in overviews of healthcare interventions, 2) to identify challenges involved when using AMSTAR in overviews and to develop potential decision rules to overcome these challenges, and 3) to examine the potential impact of considering methodological quality when making inclusion decisions in overviews. To achieve the above objectives, we examined AMSTAR assessments, inter-rater reliability of AMSTAR, the association between AMSTAR assessments and inter-rater reliability, and the association between AMSTAR assessments and results and conclusions of SRs, for both Cochrane and non-Cochrane SRs.

## Methods

### Sample selection

This descriptive study used a convenience sample of seven overviews of healthcare interventions that were selected from overviews conducted by the Alberta Research Centre for Health Evidence between 2010 to 2016. These overviews examined questions related to the efficacy or effectiveness of multiple interventions for preventing or treating clinical conditions related to pediatric health [22–28]. For each overview topic, all published English-language Cochrane and non-Cochrane SRs that met the overview’s inclusion criteria were identified from the reference list of the published overview and included in the study sample. All seven overviews included Cochrane SRs, and four also included non-Cochrane SRs. For the three overviews that did not include non-Cochrane SRs [23–25], we conducted additional literature searches to locate and include relevant non-Cochrane SRs. The literature searches were conducted by an information specialist using the inclusion criteria and search dates from each overview. Screening and inclusion were conducted independently by two reviewers, with discrepancies resolved by consensus or third party adjudication. For feasibility, we restricted the scope of one overview topic by population (outpatients only) [24]. Search strategies for all overview topics are available in published overviews and upon request.

### AMSTAR assessments

Two reviewers used the AMSTAR tool to independently assess the methodological quality of each SR included in the sample. Each of the eleven questions in the AMSTAR tool was answered “yes”, “no”, “can’t answer”, or “unable to assess”, and discrepancies between reviewers for individual AMSTAR questions were resolved via consensus or third party adjudication. In accordance with other empirical studies assessing measurement properties of AMSTAR [20, 21, 29–32], all items scoring “yes” received one point, and points were summed to a maximum of eleven for each SR.

When conducting AMSTAR assessments, reviewers also independently documented any challenges or issues that arose when assessing AMSTAR in the context of the overview of interest, including which question(s) of the AMSTAR tool were impacted by each challenge and potential reasons why each challenge posed difficulties. Reviewers also independently developed decision rules that could be used to address the challenges identified. Challenges and decision rules were discussed between reviewers until agreement was reached and were then summarized narratively.

### Result and conclusion statement assessments

The following data about the results and conclusions of each included SR were extracted: the outcome data for the first outcome listed in the corresponding overview (see Table 1);﻿ and the authors’ conclusion regarding that outcome, as stated in the abstract, discussion and/or conclusion section of each SR. For SRs that did not contain results data for the overview’s first-listed outcome, data were extracted for the overview’s second or third-listed outcome, if available. For SRs that included more than one comparison, the outcome data and conclusion statement for the comparison that was listed first in the relevant overview were extracted (as a proxy for the most clinically relevant comparison). Outcome data from the SRs contained within the procedural sedation overview were not extracted, because data for the comparator group were often not available.

**Table 1:** Overview topics and their included systematic reviews

Overview topic	Author, Year (reference)	First outcome listed in overview	Number of included systematic reviews		
			Cochrane	Non-Cochrane	Total
Acute asthma	Pollock, 2017 [22]	Hospital admission	7	6	13
Acute otitis media	Oleszczuk, 2012 [23]	Pain early in therapy	6	10	16
Bronchiolitis	Bialy, 2011 [24]	Hospital admission	4	3	7
Croup	Bjornson, 2010 [25]	Clinical score	4	2	6
Eczema	Foisy, 2011 [26]	Incidence of eczema	6	19	25
Gastroenteritis	Freedman, 2013 [27]	Hospital admission	3	12	15
Procedural sedation	Hartling, 2016 [28]	Adverse effects^a^	0	13	13
Total			30	65	95

^a^We were unable to extract primary outcome data from the systematic reviews included within the procedural sedation overview because data for the comparator group were often not available

Results and conclusions from each SR were classified based on published criteria [33, 34]. Results were classified as “favourable” (*p* < 0.10 in favour of the intervention, or finding described as ‘significant’), “neutral” (*p* > 0.10, or finding described as ‘not different between groups’), or “unfavourable” (*p* < 0.10 in favour of the comparator, or finding described as ‘favouring non-intervention comparator’). Conclusions were classified as “positive-strong” (authors stated that there was clear evidence of effectiveness, and no further research was required), “positive-weak” (authors stated that there seemed to be evidence of effectiveness, but more research was required to confirm the findings), “neutral” (authors stated that there was no or insufficient evidence about whether the intervention was effective or not, and more research was required to reach a conclusion), “negative-weak” (authors stated that there seemed to be evidence against use of the intervention, but more research was required to confirm the findings), or “negative-strong” (authors stated that there was clear evidence against use of the intervention, and no further research was required). One reviewer extracted and classified results data, and a second reviewer verified the classifications. Two reviewers independently extracted and classified conclusion statements, and discrepancies were resolved by consensus.

### Data analysis

AMSTAR assessments were summarized using means and standard deviations (SD), and independent samples t-tests were used to compare Cochrane and non-Cochrane SRs. Medians and ranges were also examined. The number and percentage of positive responses per AMSTAR question were calculated, and chi square tests were used to compare Cochrane and non-Cochrane SRs. For descriptive purposes, AMSTAR assessments were divided into categories using established criteria (AMSTAR assessments of 0-3, 4-7, and 8-11) [17, 35, 36].

Inter-rater reliability for AMSTAR was calculated using the alternative chance-correlated coefficient (AC1) statistic, with 95% confidence intervals (CIs) [37, 38]. The AC1 statistic was used in place of the kappa statistic in order to overcome the limitation of the “kappa paradox”, which occurs when high agreement between reviewers results in low kappa scores [39, 40]. Interpretation of the AC1 statistic is similar to the kappa statistic: AC1 ranges from -1.00 (perfect disagreement) to 1.00 (perfect agreement), with a value of zero indicating reliability equivalent to chance. Accordingly, inter-rater reliability was classified using criteria established by Landis & Koch: “less than chance” (<0.00), “slight” (0.00 − 0.20), “fair” (0.21 − 0.40), “moderate” (0.41 − 0.60), “substantial” (0.61 − 0.80), and “almost perfect” (0.81 − 0.99) [41, 42]. An additional level of classification, “perfect” (1.00), was also added. In addition, percentage agreement, with 95% CIs, was also calculated (overall, and per AMSTAR question), and chi square tests were used to compare Cochrane and non-Cochrane SRs.

Pearson correlation coefficients were used to correlate AMSTAR assessments and inter-rater reliability for Cochrane and non-Cochrane SRs. The strength of the resulting correlations was described using established criteria as “negligible” (0.00 − 0.30), “low” (0.30 − 0.50), “moderate” (0.50 − 0.70), “high” (0.70 − 0.90), or “very high” (0.90 − 1.00) [43]. For non-Cochrane SRs, a post-hoc regression analysis using a quadratic model was also examined (with AMSTAR assessments as the independent variable). The relationships were then depicted graphically.

The distributions of result and conclusion assessments were summarized using the number and percentage of SRs obtaining each classification. Due to the ordinal nature of the data, Mann-Whitney U-tests were used to examine differences in the breakdown of result and conclusion assessments for Cochrane compared to non-Cochrane SRs, and Spearman correlation coefficients were used to correlate AMSTAR assessments with result and conclusion assessments.

A narrative summary of challenges involved in using AMSTAR in overviews was also provided, and potential solutions were described. AgreeStat 2015.5 was used to calculate AC1 statistics (Advanced Analytics LLC., Gaitherburg, MD, USA). SPSS version 23 was used to analyze numerical data (SPSS Inc., Chicago, IL, USA).

## Results

### Study sample

**Fig. 1:**
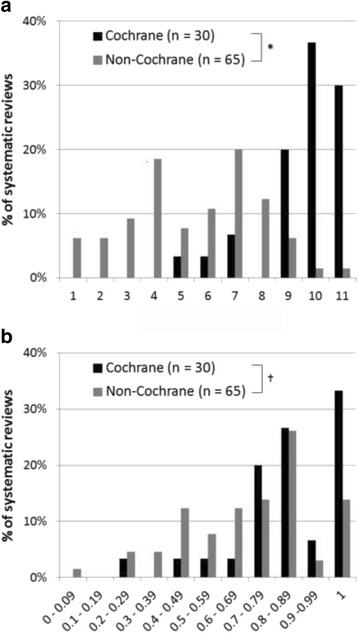
AMSTAR assessments (**a**), and inter-rater reliability (**b**), for Cochrane and non-Cochrane systematic reviews. * *p* < 0.001 in favour of Cochrane systematic reviews (independent samples t-test); † Mean inter-rater reliability was one level higher for Cochrane compared to non-Cochrane SRs (“almost perfect” vs. “substantial”)

The study sample included 95 SRs—30 Cochrane SRs and 65 non-Cochrane SRs—across seven overview topics (Table 1). A list of included SRs, along with their AMSTAR assessments, can be found in Additional file 1. The mean AMSTAR assessment (/11) for the 95 SRs was 6.8 (SD: 2.9), with ratings ranging from 1 to 11. The mean AMSTAR assessment was 9.6 (SD: 1.6) for Cochrane SRs and 5.5 (SD: 2.4) for non-Cochrane SRs. AMSTAR assessments were significantly higher for Cochrane compared to non-Cochrane SRs by a mean of 4.1 points (95% CI: 3.2, 5.1; *p* < 0.001). This pattern of results was consistent across all overview topics (with the exception of the procedural sedation topic, which had no Cochrane SRs), with mean AMSTAR assessments ranging from 1.4 points to 5.5 points higher per topic area for Cochrane compared to non-Cochrane SRs (Additional file 2, first table). Eighty-seven percent of the Cochrane SRs had AMSTAR assessments of eight or more, compared to 22% of non-Cochrane SRs. On the other hand, 22% of the non-Cochrane SRs had AMSTAR assessments of three or less, compared to 0% of Cochrane SRs (Fig. 1a). Although AMSTAR assessments were not normally distributed, mean and median assessments were very similar, and median AMSTAR assessments were higher for Cochrane compared to non-Cochrane SRs both overall and per topic area (Additional file 2, first table).

On average, Cochrane SRs received more positive responses for each of the eleven questions of the AMSTAR tool than the non-Cochrane SRs. This difference was statistically significant for eight questions (Q1-Q7, Q11; *p* ≤ 0.045). For Cochrane SRs, all eleven AMSTAR questions received positive responses more than 50% of the time (range: 53-100% positive responses per question), compared to 5/11 questions for non-Cochrane SRs (range: 14-88% positive responses per question) (Table 2, first column).

**Table 2:** Positive responses and inter-rater reliability per AMSTAR question, for Cochrane and non-Cochrane systematic reviews

AMSTAR question	Positive responses n (%)			Inter-rater reliability AC1 (95% confidence interval)		
	Cochrane (*n* = 30)	Non-Cochrane (*n* = 65)	Difference between groups (*p*-value for chi square test)	Cochrane (*n* = 30)	Non-Cochrane (*n* = 65)	Difference between groups (Landis & Koch criteria) [41]
1. Was an “a priori” design provided?	29 (96.7%)^a^	10 (15.4%)	<0.001^b^	0.93 (0.82, 1.00)	0.78 (0.63, 0.92)	“Almost perfect” vs. “substantial”^c^
2. Was there duplicate study selection and data extraction?	24 (80.0%)	21 (32.3%)	<0.001^b^	0.65 (0.36, 0.93)	0.75 (0.59, 0.91)	“Substantial” vs. “substantial”
3. Was a comprehensive literature search performed?	30 (100.0%)	42 (64.6%)	<0.001^b^	0.96 (0.89, 1.00)	0.64 (0.44, 0.83)	“Almost perfect” vs. “substantial”^c^
4. Did the authors search for reports regardless of their publication type?	27 (90.0%)	23 (35.4%)	<0.001^b^	0.85 (0.68, 1.00)	0.72 (0.55, 0.89)	“Almost perfect” vs. “substantial”^c^
5. Was a list of studies (included and excluded) provided?	30 (100.0%)	24 (36.9%)	<0.001^b^	1.00 (1.00, 1.00)	0.65 (0.47, 0.84)	“Perfect” vs. “substantial”^c^
6. Were the characteristics of the included studies provided?	30 (100.0%)	57 (87.7%)	0.045^b^	1.00 (1.00, 1.00)	0.91 (0.82, 0.99)	“Perfect” vs. “almost perfect”^c^
7. Was the scientific quality of the included studies assessed and documented?	29 (96.7%)	39 (60.0%)	<0.001^b^	0.97 (0.89, 1.00)	0.62 (0.43, 0.82)	“Almost perfect” vs. “substantial”^c^
8. Was the scientific quality of the included studies used appropriately in formulating conclusions?	26 (86.7%)	47 (72.3%)	0.12	0.79 (0.59, 0.99)	0.60 (0.40, 0.80)	“Substantial” vs. “moderate”^c^
9. Were the methods used to combine the findings of studies appropriate?	28 (93.3%)	55 (84.6%)	0.23	0.84 (0.66, 1.00)	0.69 (0.52, 0.87)	“Almost perfect” vs. “substantial”^c^
10. Was the likelihood of publication bias assessed?	16 (53.3%)	30 (46.2%)	0.52	0.67 (0.39, 0.95)	0.71 (0.53, 0.88)	“Substantial” vs. “substantial”
11. Was the conflict of interest stated?	19 (63.3%)	9 (13.9%)	<0.001^b^	0.75 (0.51, 1.00)	0.65 (0.46, 0.84)	“Substantial” vs. “substantial”

^a^One Cochrane systematic review did not have a protocol for reasons explained in the “Notes” section of the manuscript (Kramer MS, Kakuma R. Optimal duration of exclusive breastfeeding. Cochrane Database Syst Rev. 2002(1):CD003517); ^b^Significant in favour of Cochrane systematic reviews; ^c^ Inter-rater reliability for Cochrane systematic reviews was at least one level higher

### Inter-rater reliability

The mean inter-rater reliability between reviewers for the 95 included SRs, as classified using the Landis & Koch levels of classification [41], was “substantial” (AC1: 0.74; 95% CI: 0.70, 0.79), with inter-rater reliability per SR ranging from “slight” (AC1: 0.09) to “perfect” (AC1: 1.00). The mean inter-rater reliability was one level higher for Cochrane compared to non-Cochrane SRs: “almost perfect” for Cochrane SRs (AC1: 0.84; 95% CI: 0.77, 0.91) compared to “substantial” for non-Cochrane SRs (AC1: 0.69; 95% CI: 0.64, 0.75) (Fig. 1b). For the six overview topics that included both Cochrane and non-Cochrane SRs, mean inter-rater reliability ranged from 0.05-0.45 points higher per topic area for Cochrane compared to non-Cochrane SRs, and was at least one level higher for Cochrane SRs for two of the six overview topics (Additional file 2, first table). The same pattern of results was observed when examining percentage agreement; namely, agreement was higher for Cochrane compared to non-Cochrane SRs both overall, and per topic area (Additional file 2, first table).

Inter-rater reliability for the eleven individual questions of the AMSTAR tool ranged from “substantial” (Q2, Q8, Q10, Q11) to “perfect” (Q5, Q6) for the Cochrane SRs, and from “moderate” (Q8) to “almost perfect” (Q6) for the non-Cochrane SRs. Inter-rater reliability was at least one level higher for Cochrane compared to non-Cochrane SRs for 8/11 questions (Q1, Q3-Q9) (Table 2, second column). A similar pattern was observed when examining percentage agreement between reviewers: Cochrane compared to non-Cochrane SRs had higher agreement for 9/11 questions (Q1, Q3-Q9, Q11), and this difference was significant for 3/11 questions (Q3, Q5, Q7) (Additional file 2, second table).

### Challenges involved when using AMSTAR in overviews, and potential decision rules

Four main challenges were identified when assessing AMSTAR in the context of overviews. These four challenges primarily affected the AMSTAR questions concerned with quality assessments and data extraction and analysis (i.e., Q5-Q10). Each challenge is described below, along with the decision rule (and rationale) that was developed to help address each challenge (Table 3).

**Table 3:** Description of challenges identified when using AMSTAR in overviews, with corresponding recommendations

Challenge	Domain(s) affected	Explanation	Decision rule	Rationale
Many non-Cochrane SRs provided limited detail when reporting the characteristics and quality of their included pri﻿mary studies.	Q6: Were the characteristics of the included studies provided? Q7: Was the scientific quality of the included studies assessed and documented?	Q6: Some SRs presented only aggregate study characteristics; others provided insufficient detail about the populations, interventions, comparators, outcome assessments, and/or study settings. Q7: Non-Cochrane SRs used various quality assessment tools including the Cochrane Risk of Bias tool [51], the Jadad tool [75], and additional lesser-known tools. The Risk of Bias tool was often applied inconsistently across SRs, with different SRs assessing and reporting different domains.	Award point(s) only if the amount and quality of information reported in the SR is sufficient for use at the overview level.	Overview authors rely upon the primary study information as it is reported in the included SRs, and overview quality may be compromised due to in﻿adequate reporting of SRs.
Some SRs that examined the same interventions for the same disorder analyzed outcome data differently and/or came to different conclusions.	Q8: Was the scientific quality of the included studies used appropriately in formulating conclusions? Q9: Were the methods used to combine the findings of studies appropriate?	There were several instances where one SR conducted meta-analyses while another SR examining the same intervention for the same disorder presented narrative summaries only. In several instances, these SRs also reached different conclusions.	Award point(s) if authors provide appropriate justification for why they chose their method of data analysis and/or how they came to a particular conclusion.	It may not be possible to objectively determine whether the conclusions or methods of analysis of one SR we﻿re more appropriate or valid than thos﻿e in another, similar SR. It is more objective to examine the authors’ justification for whichever decisions were made.
Some SRs were broader in scope than the overview’s clinical question, meaning that some primary studies included in the SR were excluded from the overview.	Q5: Was a list of studies (included and excluded) provided? Q6, Q7, Q8, Q9: See above. Q10: Was the likelihood of publication bias assessed?	For both Cochrane and non-Cochrane SRs, there were many instances where the scope of the SR was broader than that of the overview. For example, an overview that is restricted to children only will aim to exclude adult data contained within relevant SRs.	Assess quality of the SRs overall; do not try to “piece apart” the SRs to assess only the relevant studies.	It is important to capture information about the conduct of the SR as a whole; attempting to isolate only the primary studies of interest is unnecessarily difficult.
Some non-Cochrane SRs, such as those produced for government or research organizations, searched for and included other SRs as well as primary studies. It was very difficult to assess the quality of non-Cochrane (‘original’) SRs that also included other (‘embedded’) SRs.	Q5, Q6, Q7, Q8, Q9, Q10: See above.	The original non-Cochrane SRs often did not provide sufficient information about their embedded SRs (or the studies contained within their embedded SRs). This scenario also raises additional questions for which there are no adequate answers. For example, would the original SR be awarded a point for Q5 if it did not contain a list of the studies that were included and excluded in each of its embedded SRs?	Assess the embedded SRs for inclusion into the overview. If any of them meet the inclusion criteria, obtain and refer to the full-text of these SRs and treat them as independent publications (in place of using the descriptions provided in the original SR).	It is likely not possible, nor desirable, to integrate the embedded SRs with the primary studies included in the original SR.

First, many non-Cochrane SRs provided limited detail when reporting the characteristics and evaluating the quality of their included primary studies. This often made it difficult to determine whether certain AMSTAR criteria were met, and it was unclear whether deficiencies in SRs were related to methodological quality or reporting. Overview authors rely upon the information reported in the included SRs when conducting their overview; therefore, we recommend awarding points only if the amount and quality of information reported in the SR is sufficient for use at the overview level.

Second, some SRs that examined the same interventions for the same disorder analyzed outcome data in different ways and/or reached different conclusions. It was difficult to determine whether SR methods were appropriate when different SRs used different methodologies, and we were uncertain whether multiple similar SRs should be compared against each other when conducting AMSTAR assessments. In these instances, it may not be possible to objectively determine which conclusions or methods of analysis are most appropriate or valid; therefore, we recommend awarding points only if SR authors provide appropriate justification for why they chose a certain method of analysis and/or why they reached a certain conclusion.

Third, many SRs were broader in scope than the clinical question posed in the overview, meaning that not all primary studies included in the SRs were subsequently included in the overview. Reviewers were unsure whether to assess the quality of the SRs in their entirety or whether to assess the quality of only those components of the SRs that were relevant to the overview topic. However, attempting to isolate only the components of interest in SRs was unnecessarily difficult and time-consuming, and we agreed that it was important to capture information about the conduct of the SRs as a whole. Therefore, we recommend that overview authors assess the quality of the overall SRs, without trying to “piece apart” only those components that are relevant to the overview topic.

Lastly, difficulties were encountered when assessing the quality of non-Cochrane SRs that included both primary studies and other SRs. It was unclear whether and how to assess the quality of the SRs that were embedded within the original SRs, and we were uncertain whether the AMSTAR assessments of the original SRs should be affected by the quality of the embedded SRs. When conducting AMSTAR assessments, we found that it was often not possible, nor desirable, to integrate the quality of the embedded SRs into the AMSTAR assessments of the original SRs. Therefore, when SRs include both primary studies and other embedded SRs, we recommend that overview authors treat each embedded SR as an independent publication by retrieving and assessing the full text of that SR for inclusion into the overview. The AMSTAR assessments for the overview can then proceed as usual by assessing the quality of each included SR separately.

### Association between AMSTAR assessments and inter-rater reliability

**Fig. 2:**
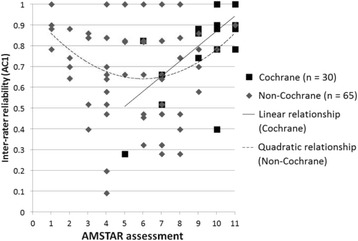
Pearson correlation between AMSTAR assessments and inter-rater reliability, for Cochrane and non-Cochrane systematic reviews. Linear relationship (Cochrane): *p* < 0.001; Quadratic relationship (non-Cochrane): *p* = 0.09

For Cochrane SRs, there was a significant positive linear correlation of “moderate” strength between AMSTAR assessments and inter-rater reliability (AC1), *r*(29) = 0.62, *p* < 0.001. Thus, inter-rater reliability increased as quality of Cochrane SRs increased. There was no evidence of a linear correlation between AMSTAR assessments and inter-rater reliability for non-Cochrane SRs (*p* = 0.38). However, visual examination of the scatterplot (Fig. 2) suggested a quadratic (curvilinear) relationship, with higher inter-rater reliability for non-Cochrane SRs that received both lower and higher assessments and lower inter-rater reliability for non-Cochrane SRs that received moderate assessments. Therefore, a quadratic model was examined. Though not statistically significant (*p* = 0.09), results suggest that inter-rater reliability may be lower for non-Cochrane SRs with moderate AMSTAR assessments and higher for non-Cochrane SRs with lower and higher ratings (Fig. 2).

### Association between AMSTAR assessments and results and conclusions of systematic reviews

There was no significant difference in the distribution of the results assessments for Cochrane compared to non-Cochrane SRs (*p* = 0.14) (Table 4). There was also no significant evidence of correlation between AMSTAR assessments and the direction of effect for the main result of each SR when looking at all SRs combined (*p* = 0.53), Cochrane SRs only (*p* = 0.30), or non-Cochrane SRs only (*p* = 0.72).

**Table 4:** Distribution of result and conclusion assessments for Cochrane and non-Cochrane systematic reviews

Result and conclusion assessments	Distribution of responses n (%)		Difference between groups (*p*-value for Mann-Whitney U-test)
	Cochrane systematic reviews (*n* = 28^a^)	Non-Cochrane systematic reviews (*n* = 43^a^)	
Results			
Unfavourable	4 (14.3%)	0 (0.0%)	0.14
Neutral	14 (50.0%)	23 (53.5%)	
Favourable	10 (35.7%)	20 (46.5%)	
Conclusions			
Negative-Strong	6 (21.4%)	1 (2.3%)	0.035^b^
Negative-Weak	6 (21.4%)	7 (16.3%)	
Neutral	3 (10.7%)	7 (16.3%)	
Positive-Weak	7 (25.0%)	13 (30.2%)	
Positive-Strong	6 (21.4%)	15 (34.9%)	

^a^Twenty-four systematic reviews (2 Cochrane, 22 non-Cochrane) were excluded from this analysis because they did not contain relevant outcome data; ^b^Conclusions of Cochrane systematic reviews were more likely to be “negative” and conclusions of non-Cochrane systematic reviews were more likely to be “positive”

The data indicated a significant difference in the distribution of the conclusion assessments for Cochrane compared to non-Cochrane SRs (*p* = 0.035). Specifically, Cochrane SRs reported significantly more “negative” conclusions, whereas non-Cochrane SRs reported significantly more “positive” conclusions (Table 4). Despite these group differences, AMSTAR assessments were not correlated with the direction and strength of effect for the main conclusion of each SR when looking at all SRs combined (*p* = 0.17), Cochrane SRs only (*p* = 0.68), or non-Cochrane SRs only (*p* = 0.80).

## Discussion

The current study used a convenience sample of 95 SRs included across seven overview topics to provide empirical evidence on issues surrounding quality assessments of SRs in overviews. This study found that AMSTAR assessments and inter-rater reliability were higher for Cochrane compared to non-Cochrane SRs; these results were consistent within each overview topic and for many of the individual questions of the AMSTAR tool. Minor challenges were encountered when assessing quality of SRs in the context of overviews, but decision rules were developed and recommendations for overview authors were provided. Results also suggested that inter-rater reliability of Cochrane and non-Cochrane SRs may be lower for SRs with moderate AMSTAR assessments and higher for SRs that were assessed as strong; inter-rater reliability may also be higher for non-Cochrane SRs assessed as weak. Consistent with a previous study [33], we found that the conclusions, but not the results, of Cochrane and non-Cochrane SRs differed systematically; however, the current study found no evidence that AMSTAR assessments were correlated with results or conclusions of SRs.

Taken together, the results of the current study suggest that AMSTAR is a useful tool for assessing the quality of Cochrane and non-Cochrane SRs in overviews. Authors should be aware of some minor challenges they may face when applying AMSTAR in overviews. Specifically, there may be deficiencies in the reporting of some SRs; SRs examining similar topics may sometimes make different methodological decisions; the scope of some SRs may differ from, or be broader than, the scope of the overview; and some non-Cochrane SRs may include other SRs as well as primary studies. We recommend that overview authors use *a priori* decision rules, such as those presented in Table 4, to circumvent these challenges and help ensure consistent judgments across reviewers. In addition, Cochrane currently recommends that quality assessments of SRs in overviews be conducted independently, in duplicate, with a process for consensus [3], and the results of this study provide empirical evidence to support this recommendation. Specifically, Cochrane SRs showed some variation in AMSTAR assessments and inter-rater reliability, and non-Cochrane SRs had lower AMSTAR assessments and inter-rater reliability combined with higher variation for both of these variables. To promote transparency, we also recommend that overview authors provide breakdowns of individual AMSTAR questions for all included SRs.

The current study found that the AMSTAR tool can be used with high inter-rater reliability to successfully identify SRs with lower quality assessments that may be difficult to use in overviews due to gross deficiencies in conduct and reporting. This study also found that AMSTAR assessments were not correlated with results or conclusions of SRs. The lack of correlation may be due to a common criticism of AMSTAR— namely, that AMSTAR may actually assess quality of reporting as well as (or instead of) methodological quality [44–46]. Quality of reporting may not necessarily be associated with SR results and conclusions. However, reporting is closely tied to usability of SRs in overviews, since overview authors cannot effectively use SRs in overviews if the data are missing, inadequately reported, or reported inconsistently [7]. The results of this study suggest that overview authors may consider using AMSTAR assessments to guide inclusion decisions (e.g., to identify and exclude poorly conducted and/or reported SRs that may be difficult to use in overviews). Using the AMSTAR tool to inform inclusion decisions may not introduce bias into the overview process since the results and conclusions of SRs assessed as weak and strong did not differ systematically. However, overview authors should use their judgment when deciding whether or not to use results of AMSTAR assessments to guide inclusion decisions. Factors to consider include the quality of the overall body of SR evidence and the purpose of the overview. For example, overview authors may not need to include poorly conducted and reported SRs when there are adequately conducted SRs that address all main interventions and outcomes of interest. In contrast, they may choose to retain poorly conducted and reported SRs when the overall body of SR evidence is generally poor or when the purpose of the overview is to describe the complete body of SR evidence on a topic. Though overall AMSTAR assessments may obscure effects of individual questions [47], SRs that score poorly across most AMSTAR domains likely have multiple serious limitations that subsequently make them difficult to include and use in overviews. To promote transparency, overview authors should establish *a priori* decision rules indicating whether and how quality assessments will be used to inform inclusion decisions; authors should also clearly indicate in their overview which SRs (if any) were excluded based on results of quality assessments or specific methodological deficiencies.

This study identified areas where authors can enhance the conduct and/or reporting of their Cochrane and non-Cochrane SRs. Previous research shows that Cochrane SRs generally have higher methodological rigour than non-Cochrane SRs [8–10], and the results of the current study extend this finding to SRs included in a sample of overviews. Non-Cochrane SRs showed room for improvement for all but two AMSTAR domains (Q6: study characteristics; Q9: methods to combine studies). However, not all Cochrane SRs received high assessments (13% were rated between 4-7 on AMSTAR), and two AMSTAR domains (Q10: publication bias; Q11: conflicts of interest) showed considerable room for improvement. This study also found that inter-rater reliability varied in conjunction with both AMSTAR assessments and type of SR (Cochrane, non-Cochrane). Inter-rater reliability was lower for SRs that obtained moderate AMSTAR assessments (as opposed to very weak or strong assessments); it was also lower for non-Cochrane SRs both overall and for many of the individual AMSTAR domains (e.g., Q3: search strategy; Q7: scientific quality; Q8: formulating conclusions). This may be because discrete “yes/no” judgments become more difficult when quality and/or reporting is mediocre, or when only some criteria for multi-part questions are addressed in the SR. The variable reporting of non-Cochrane SRs, combined with limits on manuscript length, may also contribute to difficulties conducting AMSTAR assessments. Adhering to accepted standards of conduct and reporting, such as the methods guidance contained within *The Cochrane Handbook for Systematic Reviews of Interventions* [48] and the Preferred Reporting Items for Systematic reviews and Meta-Analysis (PRISMA) reporting guidelines [49], can help ensure adequate quality and reporting of SRs (and may also increase the inter-rater reliability of quality assessments for SRs). This could, in turn, make SRs easier to assess, include, and use in overviews.

The current study used a convenience sample of overview topics that met specific inclusion criteria and shared certain characteristics (e.g., all overviews were conducted by our research group and examined interventions for disorders related to pediatric health). Though the researchers conducting this study were authors of all included overviews, they were only authors of three of the 95 included SRs, and duplicate independent quality assessments were conducted to mitigate the potential for reviewer bias. It is possible that the overview topics selected for this study may have influenced the results to some extent; however, to increase the generalizability of the knowledge gained from this study, overviews were selected that examined a range of populations (e.g., infants, children, adolescents), interventions (e.g., pharmacological, non-pharmacological), comparators (e.g., placebo, active comparators), research questions (e.g., prevention, treatment), and topic areas (e.g., acute respiratory infections, gastrointestinal diseases, skin disorders). In addition, we found that the AMSTAR assessments obtained for the SRs in our sample of seven overviews fell within the range of scores observed in a broader sample of overviews (all relevant overviews identified by [4] and [5] and contained within issue 12, 2016 of the CDSR). Our inter-rater reliability assessments were also similar to published data on agreement for AMSTAR [50]. Thus, the results of the current study, and subsequent recommendations for quality assessment of SRs in overviews, may generalize to a range of overviews examining healthcare interventions. However, results and recommendations should not be generalized to overviews that address broader or different clinical questions (e.g., diagnostic test accuracy, prognostic, or qualitative overviews).

It should be noted that there is debate surrounding whether or not overall AMSTAR scores should be calculated. The developers of AMSTAR addressed this concern by ensuring (through statistical analysis) that the component questions do not overlap and by validating the overall score against an external standard. Thus, they concluded that the overall score is meaningful [21]. However, overall quality scores assume that all questions are equal (which can be difficult to justify) [50, 51], summing individual items may artificially increase the precision of the assessment, and studies have shown that incorporating overall quality of primary studies into meta-analyses can alter effect estimates in SRs [52, 53]. Despite the uncertainty regarding use of summary scores, there is a precedent for calculating and reporting overall AMSTAR assessments both in empirical studies assessing measurement properties of AMSTAR [20, 21, 29–32, 54–56] and in overviews of healthcare interventions [16, 18, 35, 36, 57–71], and incorporating overall quality of SRs into results of overviews has not been found to alter overview results [72]. Other potential limitations of AMSTAR may include difficulty meaningfully differentiating between several of the response options (“no”, “not applicable”, and “can’t answer”) and difficulty answering multi-part questions when only some criteria are met [44, 45]. There are also no questions in the AMSTAR tool examining whether appropriate methods were used in SRs to assess the quality of a body of evidence or to conduct subgroup and/or sensitivity analyses [44, 45], and in the context of overviews AMSTAR cannot capture potentially important differences in comprehensiveness and recency of searches across SRs. As previously mentioned, the AMSTAR tool may also assess aspects related to quality of reporting as opposed to methodological quality [44–46]. Despite these potential limitations, the results of this study demonstrate that reviewers can conduct AMSTAR assessments with adequate inter-rater reliability, using decision rules to help overcome some of the above-listed challenges.

In addition to AMSTAR, other quality assessment tools exist. AMSTAR 2 is currently being developed in response to feedback from users of the original AMSTAR tool [73], and the Risk Of Bias In Systematic reviews (ROBIS) tool was recently published to assess issues related to the risk of bias (as opposed to the methodological quality) of SRs [74]. Methodological research examining the reliability, validity, and feasibility of AMSTAR 2 and ROBIS in overviews would be valuable. In addition, research comparing AMSTAR, AMSTAR 2 and ROBIS on important outcomes (e.g., quality assessments, inter-rater reliability, and time to complete assessments) and across important comparisons (e.g., Cochrane vs. non-Cochrane SRs, SRs with meta-analyses vs. narrative summaries, and publication year of SRs) may provide insight into the trade-offs involved in selecting one tool over another for use in overviews.

## Conclusions

There is currently limited guidance available for researchers conducting overviews of healthcare interventions. This gap in guidance is most pronounced when examining methods for conducting the latter stages of the overview process (e.g., quality assessments and data extraction and analysis). The current study plays a role in addressing this gap in guidance. It contributes empirical evidence and recommendations regarding the use of the AMSTAR tool to assess the methodological quality of SRs in overviews. Based on the results of this study, we show that AMSTAR can be used successfully to assess the methodological quality of both Cochrane and non-Cochrane SRs included in overviews of healthcare interventions. When using AMSTAR in overviews, individual assessments should be reported for each of the eleven questions of the AMSTAR tool. Results of quality assessments of SRs can then be used alongside quality assessments of primary studies and outcome data to help contextualize the results and conclusions of overviews.

## Additional files

Additional file 1: List of included systematic reviews, along with their AMSTAR assessments. (DOCX 33 kb)

Additional file 2: Additional results data. This file includes two tables with the following data: 1) Characteristics of included systematic reviews, by topic area, and 2) Percentage agreement per AMSTAR question, for Cochrane and non-Cochrane systematic reviews. (DOCX 19 kb)

## Abbreviations

AC1: Alternative chance-correlated coefficient
AMSTAR: A measurement tool to assess systematic reviews
CDSR: Cochrane database of systematic reviews
CI: Confidence interval
PRISMA: Preferred reporting items for systematic reviews and meta-analysis
ROBIS: Risk of bias in systematic reviews
SD: Standard deviation
SR: Systematic review
