# Supplementary material for: Evaluation of AMSTAR to assess the methodological quality of systematic reviews in overviews of reviews of healthcare interventions
Source: BMC Med Res Methodol. 2017 Mar 23;17:48. doi: 10.1186/s12874-017-0325-5 (PMC5364717; doi:10.1186/s12874-017-0325-5)
Supplement: Supplementary file 1 — List of included systematic reviews, along with their AMSTAR assessments. (DOCX 33 kb) [file 12874_2017_325_MOESM1_ESM.docx]

**Additional file 1. List of included systematic reviews, along with their AMSTAR assessments**

| **First author, year [reference]** | **Topic area** | **Type of SR** | **AMSTAR assessments** | | | | | | | | | | | |
| --- | --- | --- | --- | --- | --- | --- | --- | --- | --- | --- | --- | --- | --- | --- |
|  |  |  | **Q1** | **Q2** | **Q3** | **Q4** | **Q5** | **Q6** | **Q7** | **Q8** | **Q9** | **Q10** | **Q11** | **Total** |
| Camargo 2003 [1] | Acute asthma | Cochrane | 🗸 | 🗸 | 🗸 | 🗸 | 🗸 | 🗸 | 🗸 |  | 🗸 | 🗸 |  | 9 |
| Cates 2013 [2] | Acute asthma | Cochrane | 🗸 |  | 🗸 | 🗸 | 🗸 | 🗸 | 🗸 | 🗸 | 🗸 | 🗸 |  | 9 |
| Chavasse 2002 [3] | Acute asthma | Cochrane | 🗸 |  | 🗸 |  | 🗸 | 🗸 | 🗸 |  |  |  |  | 5 |
| Everard 2005 [4] | Acute asthma | Cochrane | 🗸 |  | 🗸 |  | 🗸 | 🗸 | 🗸 | 🗸 | 🗸 |  |  | 7 |
| Griffiths 2013 [5] | Acute asthma | Cochrane | 🗸 |  | 🗸 | 🗸 | 🗸 | 🗸 | 🗸 | 🗸 | 🗸 | 🗸 |  | 9 |
| Powell 2012 [6] | Acute asthma | Cochrane | 🗸 |  | 🗸 | 🗸 | 🗸 | 🗸 | 🗸 | 🗸 | 🗸 | 🗸 | 🗸 | 10 |
| Teoh 2012 [7] | Acute asthma | Cochrane | 🗸 | 🗸 | 🗸 | 🗸 | 🗸 | 🗸 | 🗸 | 🗸 | 🗸 | 🗸 |  | 10 |
| Amirav 1997 [8] | Acute asthma | Non-Cochrane |  |  |  |  | 🗸 | 🗸 |  | 🗸 | 🗸 |  |  | 4 |
| Castro-Rodriguez 2004 [9] | Acute asthma | Non-Cochrane |  |  | 🗸 | 🗸 | 🗸 | 🗸 | 🗸 | 🗸 | 🗸 | 🗸 |  | 8 |
| Jat 2013 [10] | Acute asthma | Non-Cochrane | 🗸 | 🗸 | 🗸 | 🗸 |  | 🗸 | 🗸 | 🗸 | 🗸 | 🗸 | 🗸 | 10 |
| Rodrigo 2005 [11] | Acute asthma | Non-Cochrane |  |  | 🗸 | 🗸 |  | 🗸 | 🗸 | 🗸 | 🗸 | 🗸 |  | 7 |
| Rodrigo 2006 [12] | Acute asthma | Non-Cochrane |  |  | 🗸 | 🗸 |  | 🗸 | 🗸 | 🗸 | 🗸 | 🗸 |  | 7 |
| Shan 2013 [13] | Acute asthma | Non-Cochrane |  |  | 🗸 |  |  | 🗸 | 🗸 | 🗸 | 🗸 | 🗸 |  | 6 |
| Coleman 2008 [14] | Acute otitis media | Cochrane | 🗸 | 🗸 | 🗸 | 🗸 | 🗸 | 🗸 | 🗸 | 🗸 | 🗸 | 🗸 | 🗸 | 11 |
| Foxlee 2006 [15] | Acute otitis media | Cochrane | 🗸 | 🗸 | 🗸 | 🗸 | 🗸 | 🗸 | 🗸 | 🗸 | 🗸 |  | 🗸 | 10 |
| Kozyrskyj 2010 [16] | Acute otitis media | Cochrane | 🗸 | 🗸 | 🗸 | 🗸 | 🗸 | 🗸 | 🗸 | 🗸 | 🗸 | 🗸 | 🗸 | 11 |
| Sanders 2004 [17] | Acute otitis media | Cochrane | 🗸 | 🗸 | 🗸 | 🗸 | 🗸 | 🗸 | 🗸 |  | 🗸 |  | 🗸 | 9 |
| Spurling 2007 [18] | Acute otitis media | Cochrane | 🗸 | 🗸 | 🗸 | 🗸 | 🗸 | 🗸 | 🗸 | 🗸 | 🗸 |  | 🗸 | 10 |
| Thanaviratananich 2008 [19] | Acute otitis media | Cochrane | 🗸 | 🗸 | 🗸 | 🗸 | 🗸 | 🗸 | 🗸 | 🗸 | 🗸 |  | 🗸 | 10 |
| Courter 2010 [20] | Acute otitis media | Non-Cochrane |  | 🗸 | 🗸 |  | 🗸 | 🗸 | 🗸 | 🗸 | 🗸 | 🗸 |  | 8 |
| Damoiseaux 1998 [21] | Acute otitis media | Non-Cochrane |  |  | 🗸 | 🗸 |  | 🗸 |  | 🗸 |  |  |  | 4 |
| Del Mar 1997 [22] | Acute otitis media | Non-Cochrane |  |  | 🗸 |  |  | 🗸 | 🗸 |  | 🗸 |  | 🗸 | 5 |
| Gulani 2009 [23] | Acute otitis media | Non-Cochrane |  |  | 🗸 | 🗸 | 🗸 | 🗸 | 🗸 | 🗸 | 🗸 | 🗸 | 🗸 | 9 |
| Ioannidis 2001 [24] | Acute otitis media | Non-Cochrane |  |  | 🗸 |  | 🗸 | 🗸 | 🗸 | 🗸 | 🗸 | 🗸 | 🗸 | 8 |
| Rahlfs 1996 [25] | Acute otitis media | Non-Cochrane |  |  |  |  |  | 🗸 |  |  |  |  |  | 1 |
| Rosenfeld 1994 [26] | Acute otitis media | Non-Cochrane | 🗸 | 🗸 |  |  |  |  | 🗸 | 🗸 |  |  |  | 4 |
| Rovers 2006 [27] | Acute otitis media | Non-Cochrane |  |  |  |  | 🗸 | 🗸 |  |  | 🗸 | 🗸 | 🗸 | 5 |
| Shekelle 2010 [28] | Acute otitis media | Non-Cochrane | 🗸 | 🗸 | 🗸 | 🗸 | 🗸 | 🗸 | 🗸 | 🗸 | 🗸 | 🗸 | 🗸 | 11 |
| Vouloumanou 2009 [29] | Acute otitis media | Non-Cochrane |  |  | 🗸 |  |  | 🗸 | 🗸 | 🗸 | 🗸 |  | 🗸 | 6 |
| Fernandes 2010 [30] | Bronchiolitis | Cochrane | 🗸 | 🗸 | 🗸 | 🗸 | 🗸 | 🗸 | 🗸 | 🗸 | 🗸 | 🗸 | 🗸 | 11 |
| Gadomski 2010 [31] | Bronchiolitis | Cochrane | 🗸 | 🗸 | 🗸 | 🗸 | 🗸 | 🗸 | 🗸 | 🗸 | 🗸 | 🗸 |  | 10 |
| Hartling 2011 [32] | Bronchiolitis | Cochrane | 🗸 | 🗸 | 🗸 | 🗸 | 🗸 | 🗸 | 🗸 | 🗸 | 🗸 | 🗸 | 🗸 | 11 |
| Zhang 2008 [33] | Bronchiolitis | Cochrane | 🗸 | 🗸 | 🗸 | 🗸 | 🗸 | 🗸 | 🗸 | 🗸 | 🗸 | 🗸 |  | 10 |
| Flores 1997 [34] | Bronchiolitis | Non-Cochrane |  |  |  | 🗸 |  | 🗸 | 🗸 | 🗸 | 🗸 |  |  | 5 |
| Kellner 1996 [35] | Bronchiolitis | Non-Cochrane |  | 🗸 | 🗸 | 🗸 | 🗸 | 🗸 | 🗸 |  |  |  |  | 6 |
| King 2004 [36] | Bronchiolitis | Non-Cochrane |  |  | 🗸 | 🗸 |  |  |  |  | 🗸 | 🗸 |  | 4 |
| Bjornson 2011 [37] | Croup | Cochrane | 🗸 | 🗸 | 🗸 | 🗸 | 🗸 | 🗸 | 🗸 | 🗸 | 🗸 | 🗸 | 🗸 | 11 |
| Moore 2006 [38] | Croup | Cochrane | 🗸 | 🗸 | 🗸 | 🗸 | 🗸 | 🗸 |  |  | 🗸 |  |  | 7 |
| Russell 2011 [39] | Croup | Cochrane | 🗸 | 🗸 | 🗸 | 🗸 | 🗸 | 🗸 | 🗸 | 🗸 | 🗸 | 🗸 | 🗸 | 11 |
| Vorweck 2010 [40] | Croup | Cochrane | 🗸 | 🗸 | 🗸 | 🗸 | 🗸 | 🗸 | 🗸 | 🗸 | 🗸 |  |  | 9 |
| Griffin 2000 [41] | Croup | Non-Cochrane |  | 🗸 | 🗸 | 🗸 | 🗸 | 🗸 | 🗸 | 🗸 | 🗸 | 🗸 |  | 9 |
| Kairys 1989 [42] | Croup | Non-Cochrane |  |  |  |  |  | 🗸 | 🗸 |  | 🗸 | 🗸 |  | 4 |
| Kramer 2002 [43] | Eczema | Cochrane |  |  | 🗸 | 🗸 | 🗸 | 🗸 | 🗸 | 🗸 |  |  |  | 6 |
| Kramer 2006 [44] | Eczema | Cochrane | 🗸 | 🗸 | 🗸 | 🗸 | 🗸 | 🗸 | 🗸 | 🗸 | 🗸 | 🗸 | 🗸 | 11 |
| Osborn 2006a [45] | Eczema | Cochrane | 🗸 | 🗸 | 🗸 |  | 🗸 | 🗸 | 🗸 | 🗸 | 🗸 |  | 🗸 | 9 |
| Osborn 2006b [46] | Eczema | Cochrane | 🗸 | 🗸 | 🗸 | 🗸 | 🗸 | 🗸 | 🗸 | 🗸 | 🗸 |  | 🗸 | 10 |
| Osborn 2007a [47] | Eczema | Cochrane | 🗸 | 🗸 | 🗸 | 🗸 | 🗸 | 🗸 | 🗸 | 🗸 | 🗸 |  | 🗸 | 10 |
| Osborn 2007b [48] | Eczema | Cochrane | 🗸 | 🗸 | 🗸 | 🗸 | 🗸 | 🗸 | 🗸 | 🗸 | 🗸 |  | 🗸 | 10 |
| Alexander 2010 [49] | Eczema | Non-Cochrane |  |  | 🗸 |  |  | 🗸 |  | 🗸 | 🗸 | 🗸 |  | 5 |
| Anandan 2009 [50] | Eczema | Non-Cochrane | 🗸 | 🗸 | 🗸 | 🗸 |  | 🗸 | 🗸 | 🗸 | 🗸 | 🗸 |  | 9 |
| Dangour 2010 [51] | Eczema | Non-Cochrane | 🗸 | 🗸 | 🗸 |  | 🗸 | 🗸 |  | 🗸 | 🗸 | 🗸 |  | 8 |
| Ernst 2002 [52] | Eczema | Non-Cochrane |  |  | 🗸 |  |  |  |  |  |  |  |  | 1 |
| Flohr 2005 [53] | Eczema | Non-Cochrane |  |  |  | 🗸 |  | 🗸 |  | 🗸 | 🗸 | 🗸 |  | 5 |
| Gdalevich 2001 [54] | Eczema | Non-Cochrane |  | 🗸 |  |  |  | 🗸 | 🗸 | 🗸 | 🗸 | 🗸 |  | 6 |
| Hanifin 2003 [55] | Eczema | Non-Cochrane |  |  | 🗸 |  |  | 🗸 | 🗸 |  |  |  |  | 3 |
| Hill 2007 [56] | Eczema | Non-Cochrane |  |  |  | 🗸 | 🗸 | 🗸 | 🗸 | 🗸 | 🗸 |  |  | 6 |
| Hoare 2000 [57] | Eczema | Non-Cochrane |  | 🗸 | 🗸 | 🗸 | 🗸 | 🗸 | 🗸 | 🗸 | 🗸 |  |  | 8 |
| Ip 2007 [58] | Eczema | Non-Cochrane |  |  | 🗸 |  | 🗸 | 🗸 | 🗸 | 🗸 | 🗸 |  | 🗸 | 7 |
| Kremmyda 2009 [59] | Eczema | Non-Cochrane |  |  |  |  |  | 🗸 |  |  |  |  |  | 1 |
| Langan 2007 [60] | Eczema | Non-Cochrane |  |  |  |  |  | 🗸 |  | 🗸 | 🗸 | 🗸 |  | 4 |
| Lee 2008 [61] | Eczema | Non-Cochrane |  |  | 🗸 |  |  | 🗸 | 🗸 |  | 🗸 |  |  | 4 |
| Muche-Borowski 2009 [62] | Eczema | Non-Cochrane |  |  | 🗸 |  |  |  |  | 🗸 |  |  |  | 2 |
| Oddy 2009 [63] | Eczema | Non-Cochrane |  |  |  |  | 🗸 |  |  | 🗸 |  |  |  | 2 |
| Schneider Chafen 2010 [64] | Eczema | Non-Cochrane | 🗸 |  | 🗸 |  | 🗸 | 🗸 | 🗸 | 🗸 | 🗸 |  |  | 7 |
| Szajewska 2010 [65] | Eczema | Non-Cochrane |  | 🗸 | 🗸 | 🗸 | 🗸 | 🗸 | 🗸 | 🗸 | 🗸 | 🗸 |  | 9 |
| Tarini 2006 [66] | Eczema | Non-Cochrane |  |  | 🗸 |  |  | 🗸 |  | 🗸 |  |  |  | 3 |
| Yang 2009 [67] | Eczema | Non-Cochrane |  | 🗸 | 🗸 |  |  | 🗸 |  | 🗸 | 🗸 | 🗸 |  | 6 |
| Allen 2010 [68] | Gastroenteritis | Cochrane | 🗸 | 🗸 | 🗸 | 🗸 | 🗸 | 🗸 | 🗸 | 🗸 | 🗸 | 🗸 | 🗸 | 11 |
| Fedorowicz 2011 [69] | Gastroenteritis | Cochrane | 🗸 | 🗸 | 🗸 | 🗸 | 🗸 | 🗸 | 🗸 | 🗸 | 🗸 |  | 🗸 | 10 |
| Hartling 2006 [70] | Gastroenteritis | Cochrane | 🗸 | 🗸 | 🗸 | 🗸 | 🗸 | 🗸 | 🗸 | 🗸 | 🗸 | 🗸 | 🗸 | 11 |
| Chmielewska 2008 [71] | Gastroenteritis | Non-Cochrane |  | 🗸 |  |  | 🗸 | 🗸 | 🗸 | 🗸 | 🗸 | 🗸 |  | 7 |
| DeCamp 2008 [72] | Gastroenteritis | Non-Cochrane |  | 🗸 |  | 🗸 |  | 🗸 | 🗸 | 🗸 | 🗸 | 🗸 |  | 7 |
| Dinleyici 2012 [73] | Gastroenteritis | Non-Cochrane |  |  | 🗸 | 🗸 |  | 🗸 |  | 🗸 | 🗸 | 🗸 |  | 6 |
| Fonseca 2004 [74] | Gastroenteritis | Non-Cochrane |  | 🗸 | 🗸 | 🗸 | 🗸 | 🗸 | 🗸 |  | 🗸 |  |  | 7 |
| Huang 2002 [75] | Gastroenteritis | Non-Cochrane |  |  | 🗸 | 🗸 | 🗸 | 🗸 | 🗸 | 🗸 | 🗸 | 🗸 |  | 8 |
| McFarland 2006 [76] | Gastroenteritis | Non-Cochrane | 🗸 |  | 🗸 | 🗸 |  | 🗸 | 🗸 |  | 🗸 | 🗸 |  | 7 |
| Salari 2012 [77] | Gastroenteritis | Non-Cochrane |  |  |  |  |  |  | 🗸 | 🗸 | 🗸 | 🗸 |  | 4 |
| Szajewska 2001 [78] | Gastroenteritis | Non-Cochrane | 🗸 | 🗸 | 🗸 |  | 🗸 | 🗸 | 🗸 |  | 🗸 |  |  | 7 |
| Szajewska 2007a [79] | Gastroenteritis | Non-Cochrane |  | 🗸 | 🗸 |  |  | 🗸 | 🗸 | 🗸 | 🗸 | 🗸 |  | 7 |
| Szajewska 2007b [80] | Gastroenteritis | Non-Cochrane |  | 🗸 | 🗸 |  | 🗸 | 🗸 | 🗸 | 🗸 | 🗸 | 🗸 |  | 8 |
| Szajewska 2007c [81] | Gastroenteritis | Non-Cochrane |  | 🗸 | 🗸 |  | 🗸 | 🗸 | 🗸 | 🗸 | 🗸 | 🗸 |  | 8 |
| Van Neil 2002 [82] | Gastroenteritis | Non-Cochrane |  | 🗸 | 🗸 | 🗸 | 🗸 | 🗸 |  |  | 🗸 | 🗸 |  | 7 |
| Deasy 2010 [83] | Procedural sedation | Non-Cochrane |  |  | 🗸 |  |  | 🗸 |  | 🗸 | 🗸 |  |  | 4 |
| Faddy 2005 [84] | Procedural sedation | Non-Cochrane |  |  |  |  |  | 🗸 | 🗸 | 🗸 | 🗸 |  |  | 4 |
| Green 2009 [85] | Procedural sedation | Non-Cochrane | 🗸 |  |  |  |  | 🗸 |  | 🗸 | 🗸 |  |  | 4 |
| Howes 2004 [86] | Procedural sedation | Non-Cochrane |  |  | 🗸 |  |  | 🗸 |  | 🗸 | 🗸 |  |  | 4 |
| Jameson 2011 [87] | Procedural sedation | Non-Cochrane |  |  |  |  |  | 🗸 |  |  | 🗸 |  |  | 2 |
| Lamond 2010 [88] | Procedural sedation | Non-Cochrane |  |  |  |  |  | 🗸 | 🗸 |  | 🗸 |  |  | 3 |
| Leroy 2010 [89] | Procedural sedation | Non-Cochrane |  |  | 🗸 |  |  |  |  | 🗸 | 🗸 |  |  | 3 |
| Mace 2004 [90] | Procedural sedation | Non-Cochrane |  |  |  |  |  | 🗸 | 🗸 | 🗸 | 🗸 |  |  | 4 |
| Migita 2005 [91] | Procedural sedation | Non-Cochrane |  | 🗸 | 🗸 | 🗸 | 🗸 | 🗸 |  | 🗸 | 🗸 |  |  | 7 |
| Mistry 2005 [92] | Procedural sedation | Non-Cochrane |  |  |  |  |  | 🗸 |  |  | 🗸 |  |  | 2 |
| National Clinical Guideline Center 2010 [93] | Procedural sedation | Non-Cochrane | 🗸 |  | 🗸 |  |  | 🗸 | 🗸 | 🗸 | 🗸 |  | 🗸 | 7 |
| Pedersen 2013 [94] | Procedural sedation | Non-Cochrane |  |  |  |  |  |  |  |  | 🗸 |  |  | 1 |
| Symington 2006 [95] | Procedural sedation | Non-Cochrane |  |  |  |  |  | 🗸 |  | 🗸 | 🗸 |  |  | 3 |

**References**

1. Camargo Jr C, Spooner C, Rowe B. Continuous versus intermittent beta-agonists for acute asthma. Cochrane Database Syst Rev. 2003(4):CD001115.

1. Cates C, Welsh E, Rowe B. Holding chambers (spacers) versus nebulisers for beta-agonist treatment of acute asthma. Cochrane Database Syst Rev. 2013(9):CD000052.

1. Chavasse R, Seddon P, Bara A, McKean M. Short acting beta2-agonists for recurrent wheeze in children under two years of age. Cochrane Database Syst Rev. 2002(2):CD002873.

1. Everard M, Bara A, Kurian M, N'Diaye T, Ducharme F, Mayowe V. Anticholinergic drugs forwheeze in children under the age of two years. Cochrane Database Syst Rev. 2005(3): CD001279.

1. Griffiths B, Ducharme F. Combined inhaled anticholinergics and short-acting beta2-agonists for initial treatment of acute asthma in children. Cochrane Database Syst Rev. 2013(8):CD000060.

1. Powell C, Dwan K, Milan S, Beasley R, Hughes R, Knopp-Sihota J, et al. Inhaled magnesium sulfate in the treatment of acute asthma. Cochrane Database Syst Rev. 2012(12):CD003898.

1. Teoh L, Cates C, Hurwitz M, Acworth J, van Asperen P, Chang A. Anticholinergic therapy for acute asthma in children. Cochrane Database Syst Rev. 2012(4):CD003797.
2. Amirav I, Newhouse M. Metered-dose inhaler accessory devices in acute asthma: efficacy and comparison with nebulizers: a literature review. Arch Pediatr Adolesc Med. 1997;151(9):876-82.

1. Castro-Rodriguez JA, Rodrigo GJ. Beta-agonists through metered-dose inhaler with valved holding chamber versus nebulizer for acute exacerbation of wheezing or asthma in children under 5 years of age: a systematic review with meta-analysis. J Pediatr. 2004;145(2):172-7.

1. Jat KR, Khairwa A. Levalbuterol versus albuterol for acute asthma: a systematic review and meta-analysis. Pulm Pharmacol Ther. 2013;26:239-48.

1. Rodrigo GJ, Castro-Rodriguez JA. Anticholinergics in the treatment of children and adults with acute asthma: a systematic review with meta-analysis. Thorax. 2005;60(9):740-6.
2. Rodrigo GJ, Nannini LJ. Comparison between nebulized adrenaline and beta2 agonists for the treatment of acute asthma. A meta-analysis of randomized trials. Am J Emerg Med. 2006;24(2):217-22.
3. Shan Z, Rong Y, Yang W, Wang D, Yao P, Xie J, et al. Intravenous and nebulized magnesium sulfate for treating acute asthma in adults and children: a systematic review and meta-analysis. Respir Med. 2013;107(3):321-30.
4. Coleman C, Moore M. Decongestants and antihistamines for acute otitis media in children. Cochrane Database Syst Rev. 2008(3):CD001727.
5. Foxlee R, Johansson A, Wejfalk J, Dooley L, Del Mar C. Topical analgesia for acute otitis media. Cochrane Database Syst Rev. 2006(3):CD005657.
6. Kozyrskyj A, Klassen T, Moffatt M, Harvey K. Short-course antibiotics for acute otitis media. Cochrane Database Syst Rev. 2010(9):CD001095.
7. Sanders S, Glasziou P, Del Mar C, Rovers M. Antibiotics for acute otitis media in children. Cochrane Database Syst Rev. 2004(1):CD000219.
8. Spurling G, Del Mar C, Dooley L, Foxlee R. Delayed antibiotics for respiratory infections. Cochrane Database Syst Rev. 2007(3):CD004417.
9. Thanaviratananich S, Laopaiboon M, Vatanasapt P. Once or twice daily versus three times daily amoxicillin with or without clavulanate for the treatment of acute otitis media. Cochrane Database Syst Rev. 2008(4):CD004975.
10. Courter JD, Baker WL, Nowak KS, Smogowicz LA, Desjardins LL, Coleman CI, et al. Increased clinical failures when treating acute otitis media with macrolides: a meta-analysis. Ann Pharmacother. 2010;44(3):471-8.
11. Damoiseaux R, van Balen F, Hoes A, de Melker R. Antibiotic treatment of acute otitis media in children under two years of age: evidence based? Br J Gen Pract. 1998;48(437):1861-4.
12. Del Mar C, Glasziou P, Hayem M. Are antibiotics indicated as initial treatment for children with acute otitis media? A meta-analysis. BMJ. 1997;314(7093):1526-9.
13. Gulani A, Sachdev H. Effectiveness of shortened course (≤ 3 days) of antibiotics for treatment of acute otitis media in children: a systematic review of randomized controlled efficacy trials. Geneva, Switzerland: World Health Organization, 2009.
14. Ioannidis J, Contopoulos-Ioannidis D, Chew P, Lau J. Meta-analysis of randomized controlled trials on the comparative efficacy and safety of azithromycin against other antibiotics for upper respiratory tract infections. J Antimicrob Chemother. 2001;48(5):677-89.
15. Rahlfs V, Macciocchi A, Monti T. Brodimoprim in upper respiratory tract infections. Clin Drug Invest. 1996;11(2):65-76.
16. Rosenfeld R, Vertrees J, Carr J, Cipolle R, Uden D, Giebink G, et al. Clinical efficacy of antimicrobial drugs for acute otitis media: metaanalysis of 5400 children from thirty-three randomized trials. J Pediatr. 1994;124(3):355-67.
17. Rovers MM, Glasziou P, Appelman CL, Burke P, McCormick DP, Damoiseaux RA, et al. Antibiotics for acute otitis media: a meta-analysis with individual patient data. Lancet. 2006;368(9545):1429-35.
18. Shekelle P, Takata G, Newberry S, Coker T, Limbos M, Chan L, et al. Management of acute otitis media: update. Rockville, MD: Agency for Healthcare Research and Quality, RAND Evidence-Based Practice Center, 2010. Report No.: 11-E004.
19. Vouloumanou EK, Karageorgopoulos DE, Kazantzi MS, Kapaskelis AM, Falagas ME. Antibiotics versus placebo or watchful waiting for acute otitis media: a meta-analysis of randomized controlled trials. J Antimicrob Chemother. 2009;64(1):16-24. Epub 2009/05/21.
20. Fernandes R, Bialy L, Vandermeer B, Tjosvold L, Plint A, Patel H, et al. Glucocorticoids for acute viral bronchiolitis in infants and young children. Cochrane Database Syst Rev. 2010(10):CD004878.
21. Gadomski A, Brower M. Bronchodilators for bronchiolitis. Cochrane Database Syst Rev. 2010(12):CD001266.
22. Hartling L, Bialy L, Vandermeer B, Tjosvold L, Johnson D, Plint A, et al. Epinephrine for bronchiolitis. Cochrane Database Syst Rev. 2011(6):CD003123.
23. Zhang L, Mendoza-Sassi R, Wainwright C, Klassen T. Nebulized hypertonic saline solution for acute bronchiolitis in infants. Cochrane Database Syst Rev. 2008(4):CD006458.
24. Flores G, Horwitz RI. Efficacy of beta 2-agonists in bronchiolitis: a reappraisal and meta-analysis. Pediatrics. 1997;100(2):233-9.
25. Kellner J, Ohlsson A, Gadomski A, Wang E. Efficacy of bronchodilator therapy in bronchiolitis. A meta-analysis. Arch Pediatr Adolesc Med. 1996;150(11):1166-72.
26. King V, Viswanathan M, Bordley W, Jackman A, Sutton S, Lohr K, et al. Pharmacologic treatment of bronchiolitis in infants and children: a systematic review. Arch Pediatr Adolesc Med. 2004;158(2):127-37.
27. Bjornson C, Russell K, Vandermeer B, Durec T, Klassen T, Johnson D. Nebulized epinephrine for croup in children. Cochrane Database Syst Rev. 2011(2):CD006619.
28. Moore M, Little P. Humidified air inhalation for treating croup. Cochrane Database Syst Rev. 2006(3):CD002870.
29. Russell K, Liang Y, O'Gorman K, Johnson D, Klassen T. Glucocorticoids for croup. Cochrane Database Syst Rev. 2011(1):CD001955.
30. Vorwerk C, Coats T. Heliox for croup in children. Cochrane Database Syst Rev. 2010(2):CD006822.
31. Griffin S, Ellis S, Fitzgerald-Barron A, Rose J, Egger M. Nebulised steroid in the treatment of croup: a systematic review of randomised controlled trials. Br J Gen Pract. 2000;50:135-41.
32. Kairys S, Marsh Olmstead E, O'Connor GT. Steroid treatment of laryngotracheitis: a meta-analysis of the evidence form randomized trials. Pediatrics. 1989;83(5):683-93.
33. Kramer MS, Kakuma R. Optimal duration of exclusive breastfeeding. Cochrane Database Syst Rev. 2002(1):CD003517.
34. Kramer MS, Kakuma R. Maternal dietary antigen avoidance during pregnancy or lactation, or both, for preventing or treating atopic disease in the child. Cochrane Database Syst Rev. 2006(3):CD000133.
35. Osborn DA, Sinn JK. Formulas containing hydrolysed protein for prevention of allergy and food intolerance in infants. Cochrane Database Syst Rev. 2006(4):CD003664.
36. Osborn DA, Sinn JK. Soy formula for prevention of allergy and food intolerance in infants. Cochrane Database Syst Rev. 2006(4):CD003741.
37. Osborn DA, Sinn JK. Prebiotics in infants for prevention of allergic disease and food hypersensitivity. Cochrane Database Syst Rev. 2007(4):CD006474.
38. Osborn DA, Sinn JK. Probiotics in infants for prevention of allergic disease and food hypersensitivity. Cochrane Database Syst Rev. 2007(4):CD006475.
39. Alexander DD, Cabana MD. Partially hydrolyzed 100% whey protein infant formula and reduced risk of atopic dermatitis: a meta-analysis. J Pediatr Gastroenterol Nutr. 2010;50(4):422-30.
40. Anandan C, Nurmatov U, Sheikh A. Omega 3 and 6 oils for primary prevention of allergic disease: systematic review and meta-analysis. Allergy. 2009;64(6):840-8.
41. Dangour AD, Lock K, Hayter A, Aikenhead A, Allen E, Uauy R. Nutrition-related health effects of organic foods: a systematic review. Am J Clin Nutr. 2010;92(1):203-10.
42. Ernst E, Pittler MH, Stevinson C. Complementary/alternative medicine in dermatology: evidence-assessed efficacy of two diseases and two treatments. Am J Clin Dermatol. 2002;3(5):341-8.
43. Flohr C, Pascoe D, Williams HC. Atopic dermatitis and the 'hygiene hypothesis': too clean to be true? Br J Dermatol. 2005;152(2):202-16.
44. Gdalevich M, Mimouni D, David M, Mimouni M. Breast-feeding and the onset of atopic dermatitis in childhood: a systematic review and meta-analysis of prospective studies. J Am Acad Dermatol. 2001;45(4):520-7.
45. Hanifin JM, Van Voorhees AS, Cooper KD, Ho VC, Kang S, Krafchik BR, et al. Guidelines of care for atopic dermatitis. American Academy of Dermatology, 2003.
46. Hill DJ, Murch SH, Rafferty K, Wallis P, Green CJ. The efficacy of amino acid-based formulas in relieving the symptoms of cow's milk allergy: a systematic review. Clin Exp Allergy. 2007;37(6):808-22.
47. Hoare C, Li Wan Po A, Williams H. Systematic review of treatments for atopic eczema. Health Technol Assess. 2000;4(37):1-191.
48. Ip S, Chung M, Raman G, Chew P, Magula N, DeVine D, et al. Breastfeeding and maternal and infant health outcomes in developed countries. Rockville, MD: Agency for Healthcare Research and Quality, Tufts-New England Medical Center Evidence-Based Practice Center, 2007. Report No.: 07-E007.
49. Kremmyda LS, Vlachava M, Noakes PS, Diaper ND, Miles EA, Calder PC. Atopy risk in infants and children in relation to early exposure to fish, oily fish, or long-chain omega-3 fatty acids: a systematic review. Clin Rev Allergy Immunol. 2009 (pre-publication);41(1):36-66.
50. Langan SM, Flohr C, Williams HC. The role of furry pets in eczema: a systematic review. Arch Dermatol. 2007;143(12):1570-7.
51. Lee J, Seto D, Bielory L. Meta-analysis of clinical trials of probiotics for prevention and treatment of pediatric atopic dermatitis. J Allergy Clin Immunol. 2008;121(1):116-21.e11.
52. Muche-Borowski C, Kopp M, Reese I, Sitter H, Werfel T, Schafer T. Allergy prevention. Dtsch Arztebl Int. 2009;106(39):625-31.
53. Oddy WH. The long-term effects of breastfeeding on asthma and atopic disease. Adv Exp Med Biol. 2009;639:237-51.
54. Schneider Chafen JJ, Newberry S, Riedl M, Bravata DM, Maglione MA, Booth M, et al. Prevalence, natural history, diagnosis, and treatment of food allergy: a systematic review of the evidence. Santa Monica, CA: National Institute on Allergy and Infectious Diseases, RAND Corporation, 2010. Report No.: WR-757-1.
55. Szajewska H, Horvath A. Meta-analysis of the evidence for a partially hydrolyzed 100% whey formula for the prevention of allergic diseases. Curr Med Res Opin. 2010;26(2):423-37.
56. Tarini BA, Carroll AE, Sox CM, Christakis DA. Systematic review of the relationship between early introduction of solid foods to infants and the development of allergic disease. Arch Pediatr Adolesc Med. 2006;160(5):502-7.
57. Yang YW, Tsai CL, Lu CY. Exclusive breastfeeding and incident atopic dermatitis in childhood: a systematic review and meta-analysis of prospective cohort studies. Br J Dermatol. 2009;161(2):373-83.
58. Allen S, Martinez E, Gregorio G, Dans L. Probiotics for treating acute infectious diarrhoea. Cochrane Database Syst Rev. 2010(11):CD003048.
59. Fedorowicz Z, Jagannath V, Carter B. Antiemetics for reducing vomiting related to acute gastroenteritis in children and adolescents. Cochrane Database Syst Rev. 2011(9):CD005506.
60. Hartling L, Bellemare S, Wiebe N, Russell K, Klassen T, Craig W. Oral versus intravenous rehydration for treating dehydration due to gastroenteritis in children. Cochrane Database Syst Rev. 2006(3):CD004390.
61. Chmielewska A, Ruszczynski M, Szajewska H. Lactobacillus reuteri strain ATCC 55730 for the treatment of acute infectious diarrhoea in children: a meta-analysis of randomized controlled trials. Pediatria Wspolczesna. 2008;10(1):32-6.
62. DeCamp L, Byerley J, Doshi N, Steiner M. Use of antiemetic agents in acute gastroenteritis: a systematic review and meta-analysis. Arch Pediatr Adolesc Med. 2008;162(9):858-65.
63. Dinleyici E, Eren M, Ozen M, Yargic Z, Vandenplas Y. Effectiveness and safety of Saccharomyces boulardii for acute infectious diarrhea. Expert Opin Biol Ther. 2012;12(4):395-410.
64. Fonseca B, Holdgate A, Craig J. Enteral vs intravenous rehydration therapy for children with gastroenteritis: a meta-analysis of randomized controlled trials. Arch Pediatr Adolesc Med. 2004;158(5):483-90.
65. Huang J, Bousvaros A, Lee J, Diaz A, Davidson E. Efficacy of probiotic use in acute diarrhea in children: a meta-analysis. Dig Dis Sci. 2002;47(11):2625-34.
66. McFarland LV, Elmer GW, McFarland M. Meta-analysis of probiotics for the prevention and treatment of acute pediatric diarrhea. Int J Probiotics Prebiotics. 2006;1(1):63-76.
67. Salari P, Nikfar S, Abdollahi M. A meta-analysis and systematic review on the effect of probiotics in acute diarrhea. Inflamm Allergy Drug Targets. 2012;11(1):3-14.
68. Szajewska H, Mrukowicz J. Probiotics in the treatment and prevention of acute infectious diarrhea in infants and children: a systematic review of published randomized, double-blind, placebo-controlled trials. J Pediatr Gastroenterol Nutr. 2001;33(Suppl 2):S17-25.
69. Szajewska H, Gieruszczak-Bialek D, Dylag M. Meta-analysis: ondansetron for vomiting in acute gastroenteritis in children. Aliment Pharmacol Ther. 2007;25(4):393-400.
70. Szajewska H, Skorka A, Dylag M. Meta-analysis: Saccharomyces boulardii for treating acute diarrhoea in children. Aliment Pharmacol Ther. 2007;25(3):257-64.
71. Szajewska H, Skorka A, Ruszczynski M, Gieruszczak-Bialek D. Meta-analysis: Lactobacillus GG for treating acute diarrhoea in children. Aliment Pharmacol Ther. 2007;25(8):871-81.
72. Van Niel CW, Feudtner C, Garrison MM, Christakis DA. Lactobacillus therapy for acute infectious diarrhea in children: a meta-analysis. Pediatrics. 2002;109(4):678-84.
73. Deasy C, Babl FE. Intravenous vs intramuscular ketamine for pediatric procedural sedation by emergency medicine specialists: a review. Paediatr Anaesth. 2010;20(9):787-96.
74. Faddy SC, Garlick SR. A systematic review of the safety of analgesia with 50% nitrous oxide: can lay responders use analgesic gases in the prehospital setting? Emerg Med J. 2005;22(12):901-8.

1. Green SM, Roback MG, Krauss B, Brown L, McGlone RG, Agrawal D, et al. Predictors of airway and respiratory adverse events with ketamine sedation in the emergency department: an individual-patient data meta-analysis of 8,282 children. Ann Emerg Med. 2009;54(2):158-68.e4.

1. Howes MC. Ketamine for paediatric sedation/analgesia in the emergency department. Emerg Med J. 2004;21(3):275-80.

1. Jameson E. Question 3 Ketamine or midazolam: does it matter which? Arch Dis Child. 2011;96(1):106-8.

1. Lamond DW. Review article: Safety profile of propofol for paediatric procedural sedation in the emergency department. Emerg Med Australas. 2010;22(4):265-86.

1. Leroy PL, Schipper DM, Knape HJ. Professional skills and competence for safe and effective procedural sedation in children: recommendations based on a systematic review of the literature. Int J Pediatr. 2010;2010:934298.

1. Mace SE, Barata IA, Cravero JP, Dalsey WC, Godwin SA, Kennedy RM, et al. Clinical policy: Evidence-based approach to pharmacologic agents used in pediatric sedation and analgesia in the emergency department. Ann Emerg Med. 2004;44(4):342-77.

1. Migita R, Klein E, Garrison M. Sedation and analgesia for pediatric fracture reduction in the emergency department: a systematic review. Arch Pediatr Adolesc Med. 2006;160(1):46-51.

1. Mistry R, Nahata M. Ketamine for conscious sedation in pediatric emergency care. Pharmacotherapy. 2005;25(8):1104-11.

1. National Clinical Guideline Centre. Sedation in children and young people: sedation for diagnostic and therapeutic procedures in children and young people. London, UK: National Institute for Health and Clinical Excellence, 2010. Report No.: 112.

1. Pedersen R, Bayat A, Steen N, Jacobsson M. Nitrous oxide provides safe and effective analgesia for minor paediatric procedures - a systematic review. Dan Med J. 2013;60(6):A4627.

1. Symington L, Thakore S. A review of the use of propofol for procedural sedation in the emergency department. Emerg Med J. 2006;23(2):89-93.
