# Supplementary material for: Evaluation of AMSTAR to assess the methodological quality of systematic reviews in overviews of reviews of healthcare interventions
Source: BMC Med Res Methodol. 2017 Mar 23;17:48. doi: 10.1186/s12874-017-0325-5 (PMC5364717; doi:10.1186/s12874-017-0325-5)
Supplement: Supplementary file 2 — Additional results data. This file includes two tables with the following data: 1) Characteristics of included systematic reviews, by topic area, and 2) Percentage agreement per AMSTAR question, for Cochrane and non-Cochrane systematic reviews. (DOCX 19 kb) [file 12874_2017_325_MOESM2_ESM.docx]

**Additional file 2. Additional results data**

**Table 1.** Characteristics of included systematic reviews, by topic area

|  | **Number of included reviews** | **Years of publication (median, range)** | **AMSTAR assessments**  **(mean, standard deviation)** | **AMSTAR assessments**  **(median,**  **range)** | **Inter-rater reliability**  **(AC1, 95% confidence interval^a^)** | **Percentage agreement**  **(%, 95% confidence interval^a^)** |
| --- | --- | --- | --- | --- | --- | --- |
| **Acute asthma** | **13** | **2006 (1997-2013)** | **7.8 (2.0)** | **8.0 (4.0-10.0)** | **0.74 (0.59, 0.88)** | **84.6 (77.4, 91.8)** |
| Cochrane | 7 | 2012 (2002-2013) | 8.4 (1.8) | 9.0 (5.0-10.0) | 0.76 (0.51, 1.00) | 85.7 (73.0, 98.4) |
| Non-Cochrane | 6 | 2006 (1997-2013) | 7.0 (2.0) | 7.0 (4.0-10.0) | 0.71 (0.49, 0.93) | 83.3 (72.2, 94.5) |
| **Acute otitis media** | **16** | **2006 (1994-2010)** | **7.6 (3.1)** | **8.5 (1.0-11.0)** | **0.93 (0.88, 0.98)** | **95.5 (92.4, 98.5)** |
| Cochrane | 6 | 2008 (2004-2010) | 10.2 (0.8) | 10.0 (9.0-11.0) | 0.98 (0.92, 1.00) | 98.5 (94.6, 100.0) |
| Non-Cochrane | 10 | 2004 (1994-2010) | 6.1 (2.9) | 5.5 (1.0-11.0) | 0.90 (0.83, 0.97) | 93.6 (89.2, 98.0) |
| **Bronchiolitis** | **7** | **2008 (1996-2011)** | **8.1 (3.0)** | **10.0 (4.0-11.0)** | **0.69 (0.43, 0.94)** | **80.5 (66.4, 94.6)** |
| Cochrane | 4 | 2010 (2008-2011) | 10.5 (0.6) | 10.5 (10.0-11.0) | 0.74 (0.37, 1.00) | 81.8 (61.4, 100.0) |
| Non-Cochrane | 3 | 1997 (1996-2004) | 5.0 (1.0) | 5.0 (4.0-6.0) | 0.61 (0.00, 1.00) | 78.8 (26.6, 100.0) |
| **Croup** | **6** | **2008 (1989-2012)** | **8.3 (3.0)** | **9.0 (3.0-11.0)** | **0.76 (0.64, 0.88)** | **83.3 (76.2, 90.5)** |
| Cochrane | 4 | 2011 (2006-2012) | 9.5 (1.9) | 10.0 (7.0-11.0) | 0.78 (0.63, 0.92) | 84.1 (76.9, 91.3) |
| Non-Cochrane | 2 | 1995 (1989-2000) | 6.0 (4.2) | 6.0 (3.0-9.0) | 0.72 (0.00, 1.00) | 81.8 (0.00, 100.0) |
| **Eczema** | **25** | **2007 (2000-2010)** | **6.1 (3.0)** | **6.0 (1.0-11.0)** | **0.76 (0.68, 0.84)** | **85.8 (81.6, 90.0)** |
| Cochrane | 6 | 2006 (2002-2007) | 9.3 (1.8) | 10.0 (6.0-11.0) | 0.85 (0.76, 0.95) | 89.4 (82.2, 96.6) |
| Non-Cochrane | 19 | 2008 (2000-2010) | 5.1 (2.6) | 5.0 (1.0-9.0) | 0.73 (0.63, 0.83) | 84.7 (0.79, 0.90) |
| **Gastroenteritis** | **15** | **2007 (2001-2012)** | **7.7 (1.8)** | **7.0 (4.0-11.0)** | **0.61 (0.46, 0.76)** | **78.2 (70.6, 85.8)** |
| Cochrane | 3 | 2010 (2006-2011) | 10.7 (0.6) | 11.0 (10.0-11.0) | 0.97 (0.82, 1.00) | 97.0 (83.9, 100.0) |
| Non-Cochrane | 12 | 2007 (2001-2012) | 6.9 (1.1) | 7.0 (4.0-8.0) | 0.52 (0.38, 0.66) | 73.5 (66.8, 80.2) |
| **Procedural sedation** | **13** | **2009 (2004-2013)** | **3.7 (1.8)** | **4.0 (1.0-7.0)** | **0.65 (0.53, 0.78)** | **79.7 (72.9, 86.5)** |
| Cochrane | 0 | NA | NA | NA | NA | NA |
| Non-Cochrane | 13 | 2009 (2004-2013) | 3.7 (1.8) | 4.0 (1.0-7.0) | 0.65 (0.53, 0.78) | 79.7 (72.9, 86.5) |
| **Total** | **95** | **2007 (1989-2013)** | **6.8 (2.9)** | **7.0 (1.0-11.0)** | **0.74 (0.70, 0.79)** | **84.7 (82.3, 87.1)** |
| Cochrane | 30 | 2009 (2002-2013) | 9.6 (1.6) | 10.0 (5.0-11.0) | 0.84 (0.77, 0.91) | 89.4 (85.5, 93.3) |
| Non-Cochrane | 65 | 2007 (1989-2013) | 5.5 (2.4) | 6.0 (1.0-11.0) | 0.69 (0.64, 0.75) | 82.5 (79.5, 85.5) |

^a^ 95% confidence intervals were capped at 0.00 and 1.00 for inter-rater reliability and 0.00 and 100.0 for percent agreement.

**Table 2.** Percentage agreement per AMSTAR question, for Cochrane and non-Cochrane systematic reviews

| **AMSTAR question** | **Number of agreements**  **N (%)** | | |
| --- | --- | --- | --- |
|  | **Cochrane**  **(n = 30)** | **Non-Cochrane**  **(n = 65)** | **Difference between groups**  **(p-value for chi square test)** |
| 1. Was an "a priori" design provided? | 28 (93.3%) | 54 (83.1%) | 0.18 |
| 2. Was there duplicate study selection and data extraction? | 20 (66.7%) | 56 (86.2%) | 0.027^a^ |
| 3. Was a comprehensive literature search performed? | 29 (96.7%) | 52 (80.0%) | 0.033^b^ |
| 4. Did the authors search for reports regardless of their publication type? | 26 (86.7%) | 55 (84.6%) | 0.79 |
| 5. Was a list of studies (included and excluded) provided? | 30 (100.0%) | 53 (81.5%) | 0.012^b^ |
| 6. Were the characteristics of the included studies provided? | 30 (100.0%) | 60 (92.3%) | 0.12 |
| 7. Was the scientific quality of the included studies assessed and documented? | 29 (96.7%) | 52 (80.0%) | 0.033^b^ |
| 8. Was the scientific quality of the included studies used appropriately in formulating conclusions? | 26 (86.7%) | 51 (78.5%) | 0.34 |
| 9. Were the methods used to combine the findings of studies appropriate? | 26 (86.7%) | 52 (80.0%) | 0.43 |
| 10. Was the likelihood of publication bias assessed? | 25 (83.3%) | 55 (84.6%) | 0.87 |
| 11. Was the conflict of interest stated? | 26 (86.7%) | 50 (76.9%) | 0.27 |

^a^ Significant in favour of non-Cochrane systematic reviews; ^b^ Significant in favour of Cochrane systematic reviews.
